# HIV and concurrent sexual partnerships: modelling the role of coital dilution

**DOI:** 10.1186/1758-2652-14-44

**Published:** 2011-09-13

**Authors:** Larry Sawers, Alan G Isaac, Eileen Stillwaggon

**Affiliations:** 1 Department of Economics, American University, Washington DC, USA; 2 Department of Economics, Gettysburg College, Gettysburg, PA, USA

## Abstract

**Background:**

The concurrency hypothesis asserts that high prevalence of overlapping sexual partnerships explains extraordinarily high HIV levels in sub-Saharan Africa. Earlier simulation models show that the network effect of concurrency can increase HIV incidence, but those models do not account for the coital dilution effect (non-primary partnerships have lower coital frequency than primary partnerships).

**Methods:**

We modify the model of Eaton *et al *(*AIDS and Behavior*, September 2010) to incorporate coital dilution by assigning lower coital frequencies to non-primary partnerships. We parameterize coital dilution based on the empirical work of Morris *et al *(*PLoS ONE*, December 2010) and others. Following Eaton *et al*, we simulate the daily transmission of HIV over 250 years for 10 levels of concurrency.

**Results:**

At every level of concurrency, our focal coital-dilution simulation produces epidemic extinction. Our sensitivity analysis shows that this result is quite robust; even modestly lower coital frequencies in non-primary partnerships lead to epidemic extinction.

**Conclusions:**

In order to contribute usefully to the investigation of HIV prevalence, simulation models of concurrent partnering and HIV epidemics must incorporate realistic degrees of coital dilution. Doing so dramatically reduces the role that concurrency can play in accelerating the spread of HIV and suggests that concurrency cannot be an important driver of HIV epidemics in sub-Saharan Africa. Alternative explanations for HIV epidemics in sub-Saharan Africa are needed.

## Background

The concurrency hypothesis asserts that the high prevalence of overlapping sexual partnerships - known as concurrency or multiple concurrent partnering - explains the extraordinarily high levels of HIV in sub-Saharan Africa. For the hypothesis to be valid, concurrency must be especially effective in spreading HIV. To assess the implications of concurrency for the spread of HIV, researchers have turned to formal models.

The present paper describes a logical and empirical error - the failure to incorporate coital dilution - embedded in many well-known sexual-network models that purport to demonstrate a critical role for concurrency in spreading HIV [1-8]. We develop a simple, empirically grounded modification of a model recently published by Eaton, Hallett and Garnett [9]. The corrected model does not generate any sustainable epidemic of HIV, even at implausibly high levels of concurrency. Our results support the view that concurrency cannot be a principal driver of sub-Saharan Africa's HIV epidemics.

### Evidence of coital dilution

Coital dilution is the reduction in per-partner coital frequency that accompanies the acquisition of additional partners. A person who acquires additional partners may very well increase his or her total coital frequency, but coital dilution means that his or her average per-partner coital frequency will decline with the acquisition of each additional partner. Relevant evidence is not plentiful, but it confirms the existence of coital dilution. Morris, Epstein and Wawer [10] report survey evidence from Rakai, Uganda, and they find that reported coital frequencies in secondary partnerships are less than one-quarter those in primary partnerships. Harrison *et al *studied youth aged 15 to 24 years in KwaZulu-Natal, South Africa [11], and present the proportion of respondents who reported sex in the previous week, month and year with their two most recent partners. Sexual contact was far less frequent in secondary partnerships.

Of women interviewed in the Lesotho 2009 Demographic and Health Survey (DHS), 2.3% reported overlapping partners six months prior to the interview (calculated by the authors using a dataset supplied by MEASURE DHS - ICF Macro). Of those, nearly all (96%) reported only two partners in the previous year. Among those women, 41% reported at least weekly sex (52 or more times a year) with their most recent partner, but only 12% reported coital frequency that high with their second most recent partner. Only 9% reported six or fewer coital acts in the previous year with their most recent partner, but more than half reported sex that infrequently with their second most recent partner. Gourvenec *et al *[12] report on sexually experienced women aged 15 to 34 years in Botswana. Seventy percent of those with on-going concurrent partnerships reported sex with only one partner in the previous month. (There is also evidence of coital dilution in the US [10,13].)

A substantial literature on fertility and polygyny in sub-Saharan Africa presumes or confirms coital dilution in polygynous marriages in the region [14-16]. Many concurrent partnerships, even though not sanctioned by law, religion or custom, function as though they were polygynous unions, so that literature is relevant to a broader discussion of concurrency. Coital dilution is part of the reason why polygyny appears to protect populations from HIV [17-19]. Reniers and Tfaily's study of 20 countries in sub-Saharan Africa reports that "compared to women in monogamous unions, women in polygynous unions report lower coital frequency in all but one of the countries" [19]. Stewart *et al *find that in the Central African Republic reported coital frequency was lower among women in polygynous marriages than among monogamous women and that "coital frequency decreased significantly as the number of co-wives increased" ([20] page 531).

Coital dilution is also intuitively plausible or even obvious. Long-distance migration for extended periods, which is common in sub-Saharan Africa and often cited as a source of concurrency in the region [21,22], can lead to second partnerships that fall within the standard definition of concurrency. Nevertheless, those partnerships also resemble serial monogamy: the partner in town or at the mines only replaces the coition that the migrant might have had with a partner at home. In addition, common sense tells us that simply multiplying the number of sex acts in proportion to the number of additional partners would surpass many people's organizational and time-budgeting skills [13].

### Concurrency, coital dilution and HIV

There are two possible mechanisms by which concurrency can affect the spread of HIV: a network effect and a coital dilution effect. To determine the impact of differences in the level of concurrency on HIV transmission, one must hold constant anything else that might affect HIV incidence, including the number of partnerships in the population. More concurrency means that there are more partnerships that overlap, not more partnerships. An increase in the number of overlapping partnerships in a population of a given size with a fixed number of partnerships necessarily implies an increase in the number of individuals who have no partner. Thus, concurrency concentrates sexual activity in a subset of the population. That concentration, other things equal, could be expected to accelerate the spread of HIV. We call that the "network effect".

At the same time, concurrency reduces total coition in a population because of coital dilution, which is the lower average per-partnership coital frequency in the population at higher levels of concurrency. If the number of partnerships in a population is constant and the average coital frequency in those partnerships falls, then total coition must also fall. The lower the average coital frequency, other things equal, the slower is the sexual spread of HIV since there are fewer sex acts by which the infection can be transmitted. We call that the "coital dilution effect". The strength of the coital dilution effect on HIV, of course, depends on how rapidly average per-partner coital frequency falls with any increase in the share of overlapping partnerships among total partnerships. Whether the network effect or the coital dilution effect is stronger cannot be determined *a priori*.

### Modelling concurrency

Our model of HIV transmission in sexual networks is an adaptation of the model developed by Eaton *et al *[9], which is a modified version of Morris and Kretzschmar's model [2]. Eaton *et al *replace Morris and Kretzschmar's invariant (and excessively high) transmission rate with one that varies with the stage of infection, relying on the calculations of Hollingsworth *et al *[23], who rework data from a study by Wawer *et al *[24]. (Eaton *et al *also incorporate vital dynamics, such as births and deaths, allowing them to model epidemics for 250 years, not just for the five years considered by Morris and Kretzschmar.)

Eaton *et al *find that "with staged transmission and up to 8% [point prevalence] of individuals having concurrent partnerships, HIV fails to spread" [9]. That finding seriously undermines the concurrency hypothesis, since no country-level survey in sub-Saharan Africa using currently accepted questionnaire designs [25] has found point prevalence of concurrency for all adults higher than 8% (see Table One in [26]). Rates of concurrency exceeding 8% can be found in subpopulations within sub-Saharan African countries, but the concurrency hypothesis concerns differences between countries or groups of countries, not selected sub-national groups.

### Coital frequency in precursor models

Agent-based simulation models of sexual-network dynamics and HIV (such as Morris and Kretzschmar and Eaton *et al*) specify a daily risk of transmission in each sero-discordant partnership. Models of this genre have no per-act transmission rate or coital frequency parameters *per se *and make no explicit assumptions about the value of those variables. Nevertheless, the daily risk of infection is conceptually the product of a per-act transmission rate and coital frequency. By assuming a daily risk of infection (either fixed or stage-specific) that is common to all partnerships, Morris and Kretzschmar and Eaton *et al *have implicitly assumed that coital frequency is the same in all partnerships (or in all partnerships at any given stage of infection). See Additional File 1 for a fuller discussion of this issue.

Morris and Kretzschmar assume a fixed 0.05 daily infection risk, for which they offer no explanation and provide no citation [2]. Eaton *et al*'s daily stage-specific risk of infection is Hollingsworth *et al*'s stage-specific annual risk of infection divided by 365. A brief explanation of how they determined that annual risk of infection may be useful to the reader. To measure per-act transmission risk, one must first specify a period during which transmission takes place and then count both the number of sex acts and the number of seroconversions during that period. Dividing the number of seroconversions by the number of sex acts yields the per-act transmission rate, and dividing the number sex acts by time yields coital frequency. Hollingsworth *et al *argue that measuring the frequency of sex acts is subject to substantial reporting error, which produces a corresponding error in the per-act transmission rate [23].

Modellers, however, do not need to know the value of either of those error-prone variables. They need only a time-dependent risk of infection, which can be estimated directly from data on seroconversions over a specified period. Accordingly, Hollingsworth *et al*, and therefore Eaton *et al *never specify a value for coital frequency. Instead, they use a daily stage-specific risk of infection. Nevertheless, using the same rate for all partnerships is problematic in the presence of concurrency since it presumes the same (though unspecified) coital frequency at each stage of infection for both primary and non-primary partnerships. That presumption of the same coital frequency, however, is inconsistent with the evidence for the existence of coital dilution.

Additionally, Hollingsworth *et al *estimated their infection risk using data from Wawer *et al *[24], who studied transmission only between partners who "reported that they were monogamous (defined as having only 1 sex partner during the period of observation)" (page 1404). Eaton *et al*'s daily risk of infection is thus derived from data only from primary partnerships in which both partners reported no other partner. Nevertheless, they assign the same daily stage-specific risk of infection to both monogamous and concurrent partnerships. That is appropriate only if monogamous and concurrent partnerships have the same coital frequency, which - given the evidence for coital dilution - they do not.

We do not have any direct measure of the daily risk of infection in non-primary partnerships, but the evidence shows that coital frequencies in non-primary partnerships are much lower than in primary partnerships. We respond to this evidence by distinguishing primary and non-primary partnerships and assuming lower daily risk of infection in non-primary partnerships.

## Methods

Our sole objective in this article is to demonstrate the impact of incorporating coital dilution into a model of sexual network dynamics and HIV. We therefore select a baseline model [9] that is a substantial improvement on the model created by pioneers in the field [2]. Our model successfully replicates the baseline results under the assumption of no coital dilution. We then offer a focal simulation with 75% coital dilution, along with a sensitivity analysis.

### Critical assumptions

As noted, modelling the effects of concurrency *per se *requires holding constant the number of partnerships in the modelled population as one changes the level of concurrency. In other words, modelling an increase in the level of concurrency only reapportions partnerships within the population such that some individuals gain additional partnerships, leaving others with fewer partners or none. We follow that established practice, as do both Eaton *et al *[9] and Morris and Kretzschmar [2]. The latter say that their model is "carefully structured to ensure that concurrency is not confounded with a simple increase in the number of partnerships". Of course, a model that also increased the number of partnerships would generate a more rapid spread of HIV [3], but it could not disentangle the separate effects of increased sexual partnering in general from concurrency specifically.

Holding constant the number of partnerships is also consistent with evidence on the prevalence of partnering in sub-Saharan Africa. Supporters of the concurrency hypothesis argue that concurrency is more prevalent in the region than in countries with much lower HIV prevalence. They acknowledge, however, that the prevalence of multiple partnering in the previous year or in a lifetime is not especially high in sub-Saharan Africa [27,28], a finding that is confirmed by numerous surveys (for example, see [29]). Survey data also show that the proportion of adults with even one sexual partner is much lower in sub-Saharan Africa than in the US or Europe. For example, the prevalence of adult men reporting any female sexual partner in the previous year is 78.9% in the US and 96.3% (unweighted average) in 10 European countries, but only 67.1% (weighted average) in 18 sub-Saharan African countries (Table Two in [30], Tables 5.2 and 5.6 in [31], and Table Five in [32]). Modelling that allows the number of partnerships to rise as the level of concurrency is increased does not reflect the sub-Saharan African reality, which is characterized by a lower prevalence of partnering.

A second critical assumption follows from the first and has to do with the frequency of sex acts in the modelled population. If coital dilution is introduced into a model in which the number of partnerships is fixed, then coital frequency in the population as a whole must fall. That follows from the definition of coital dilution, which is the decline in average per-partner coital frequency with the acquisition of additional partners, that is, with an increase in concurrency. Goodreau urges holding constant the number of sex acts "so that observed epidemic differences do not simply reflect changes in coital acts" [33]. The only way to accept Goodreau's advice without increasing the number of partnerships is to disregard coital dilution.

At issue is whether or not coital dilution is an essential empirical dimension of concurrency. We argue that it is. As we have seen, the evidence in support of coital dilution is not abundant, but is unanimous, and concurrent partnering without coital dilution in any actual society is implausible. Goodreau effectively counsels us to ignore the empirical reality of concurrency and examine only the network effect of concurrency without the coital dilution effect. Ignoring coital dilution by holding total coition constant thus obscures rather than clarifies the role of concurrency in spreading HIV, and produces no results of interest or consequence.

### Parameter values and algorithm configuration

Other than incorporating coital dilution, our simulation procedures replicate those of Eaton *et al*. See Table 1 for parameter values used in our simulations. We simulate the daily sexual behaviours, infections and deaths in a population of 10,000 men and 10,000 women for 250 years. We run 100 replicates of each simulation scenario, recording the mean HIV prevalence on each day. We initialize the HIV epidemic by infecting 1% of men and 1% of women. Our focal simulation assumes 75% coital dilution, and we include a sensitivity analysis that explores various degrees of coital dilution.

**Table 1:** Parameters and parameter values used in simulations^a^

*Parameter*	*Parameter Value*
Number of males in population	10,000

Number of females in population	10,000

Partnership formation rate	0.01

Partnership dissolution probability	0.005/day

Mean partnership duration	200 days

Primary partnership transmission probability	

during primary infection	0.00732/day

during asymptomatic infection	0.00029/day

during symptomatic infection	0.00208/day

during severe AIDS infection	0.0/day

Non-primary partnership transmission probability	

in focal simulation	75% less than in primary partnerships

in sensitivity analysis	55%, 35%, 25%, 15%, 0% smaller

Duration of primary infection	88 days

Duration of asymptomatic infection	3054 days

Duration of symptomatic infection	274 days

Duration between infection and death	3723 days

Number of male seed infections	100 (1%)

Number of female seed infections	100 (1%)

^a^This table is adapted from Eaton *et al*, Table One [9] by specifying different transmission probabilities for primary and non-primary partnerships. Primary partnership transmission probabilities are taken directly from [9].

We compare our results with those of Eaton *et al*, which we replicate with 0% coital dilution. We consider the same 10 levels of concurrency, including serial monogamy, used by Eaton *et al*. We measure the level of concurrency by point prevalence. The maximum level of concurrency considered is 14%, at which point about 30% of those with partners have more than one partner and two-thirds of all partnerships in the population are concurrent. That is the maximum level considered by Eaton *et al *and by Morris and Kretzschmar [2]. It is not far from the theoretical maximum level of concurrency at which point all partnerships are concurrent.

Modifying Eaton *et al*'s model to encompass coital dilution requires changing both their algorithm and some parameter values: we must introduce lower daily risks of infection in non-primary partnerships, and we must distinguish algorithmically between primary and non-primary partnerships. We assign differential infection risks in primary and non-primary partnerships based on data from Morris, Epstein and Wawer's survey in Rakai, Uganda, in 1993 and 1994 [10], which reports median annual coition in primary and secondary partnerships for men and women separately. Based on those data, our focal simulation makes the assumption that coital frequency in secondary partnerships is 25% of that in primary partnerships, which is somewhat above the observed percentage in Morris *et al*. Lacking empirical evidence on coital frequencies in third, fourth and fifth partnerships, we assume identical coital frequencies in all non-primary partnerships.

Upon partnership formation, our model assigns partnership status as follows: if neither partner has a primary partner, a new partnership is the primary partnership. All other partnerships are designated as non-primary. Once designated as primary or non-primary, that designation remains until the partnership dissolves. Any rule of this sort is arbitrary. We do not believe that different decision rules would lead to important changes in our results, but to hedge against that possibility, we test for robustness by varying the difference in infection risk between primary and secondary partnerships.

## Results

**Figure 1:**
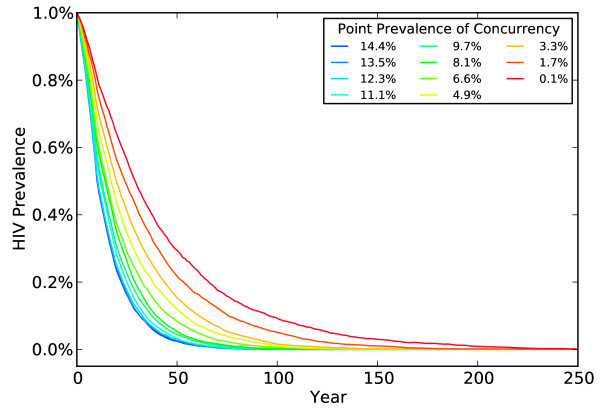
**HIV prevalence at different levels of concurrency from 0 to 250 years with coital dilution of 75%**.

In the context of the literature concerned with the effect of concurrency on HIV prevalence, the results of our focal simulation (the first one shown in Table 2; see also Figure 1) appear startling: the higher the level of concurrency, the more quickly HIV prevalence falls, and the sooner the epidemic reaches extinction. At the highest level of concurrency, HIV prevalence falls by nearly half in 10 years, as deaths from AIDS outpace incident infections. In 30 years, prevalence falls by 90%. The process of extinction is slower with serial monogamy. It takes 30 years for HIV prevalence to fall by half and nearly 100 years to fall by 90%. At intermediate levels of concurrency, the epidemic paths lie between those two extremes. Our results confirm the importance of coital dilution in understanding the impact of concurrency on HIV epidemics. Nevertheless, for reasons we will discuss, we believe that even our simulations overstate the importance of concurrency in spreading HIV.

**Table 2:** HIV prevalence at different degrees of concurrency with 75%, 55%, 35%, 25%, 15%, and 0% coital dilution at 0 to 250 years

Time	*0% Concurrency*	*3% Concurrency*	*7% Concurrency*	*10% Concurrency*	*12% Concurrency*	*14% Concurrency*
**75% coital dilution **(daily risk of infection in non-primary partnerships is 25% of primary partnership risk)						

*0 years*	1.00%	1.00%	1.00%	1.00%	1.00%	1.00%

*10 years*	0.81%	0.72%	0.64%	0.58%	0.52%	0.51%

*25 years*	0.56%	0.41%	0.29%	0.23%	0.17%	0.16%

*50 years*	0.29%	0.15%	0.08%	0.04%	0.03%	0.02%

*100 years*	0.09%	0.02%	0.01%	0.00%	0.00%	0.00%

*250 years*	0.00%	0.00%	0.00%	0.00%	0.00%	0.00%

**55% coital dilution **(daily risk of infection in non-primary partnerships is 45% of primary partnership risk)						

*0 years*	1.00%	1.00%	1.00%	1.00%	1.00%	1.00%

*10 years*	0.81%	0.75%	0.71%	0.70%	0.68%	0.70%

*25 years*	0.57%	0.45%	0.40%	0.36%	0.35%	0.37%

*50 years*	0.30%	0.21%	0.16%	0.13%	0.12%	0.14%

*100 years*	0.09%	0.04%	0.03%	0.01%	0.01%	0.02%

*250 years*	0.00%	0.00%	0.00%	0.00%	0.00%	0.00%

**35% coital dilution **(daily risk of infection in non-primary partnerships is 65% of primary partnership risk)						

*0 years*	1.00%	1.00%	1.00%	1.00%	1.00%	1.00%

*10 years*	0.81%	0.80%	0.81%	0.82%	0.85%	0.92%

*25 years*	0.56%	0.55%	0.55%	0.59%	0.64%	0.80%

*50 years*	0.30%	0.29%	0.28%	0.33%	0.41%	0.64%

*100 years*	0.09%	0.07%	0.07%	0.10%	0.18%	0.38%

*250 years*	0.00%	0.00%	0.00%	0.01%	0.02%	0.08%

**25% coital dilution **(daily risk of infection in non-primary partnerships is 75% of primary partnership risk)						

*0 years*	1.00%	1.00%	1.00%	1.00%	1.00%	***1.00%***

*10 years*	0.81%	0.81%	0.84%	0.89%	0.97%	***1.06%***

*25 years*	0.56%	0.57%	0.62%	0.71%	0.86%	***1.13%***

*50 years*	0.29%	0.32%	0.35%	0.51%	0.71%	***1.21%***

*100 years*	0.09%	0.10%	0.14%	0.25%	0.54%	***1.41%***

*250 years*	0.00%	0.00%	0.01%	0.03%	0.20%	***1.80%***

**15% coital dilution **(daily risk of infection in non-primary partnerships is 85% of primary partnership risk)						

*0 years*	1.00%	1.00%	1.00%	1.00%	***1.00%***	***1.00%***

*10 years*	0.81%	0.86%	0.88%	0.96%	***1.03%***	***1.18%***

*25 years*	0.56%	0.63%	0.70%	0.89%	***1.09%***	***1.49%***

*50 years*	0.29%	0.38%	0.51%	0.75%	***1.21%***	***2.27%***

*100 years*	0.09%	0.13%	0.25%	0.58%	***1.48%***	***4.20%***

*250 years*	0.00%	0.01%	0.03%	0.21%	***2.25%***	***8.26%***

**No coital dilution **(daily risk of infection in non-primary partnerships is the same as primary partnership risk)						

*0 years*	1.00%	1.00%	1.00%	***1.00%***	***1.00%***	***1.00%***

*10 years*	0.81%	0.87%	0.96%	***1.08%***	***1.21%***	***1.42%***

*25 years*	0.57%	0.67%	0.86%	***1.19%***	***1.64%***	***2.42%***

*50 years*	0.30%	0.42%	0.73%	***1.38%***	***2.61%***	***5.29%***

*100 years*	0.10%	0.18%	0.52%	***1.71%***	***5.37%***	***12.42%***

*250 years*	0.00%	.02%	0.19%	***2.72%***	***10.02%***	***15.70%***

**Figure 2:**
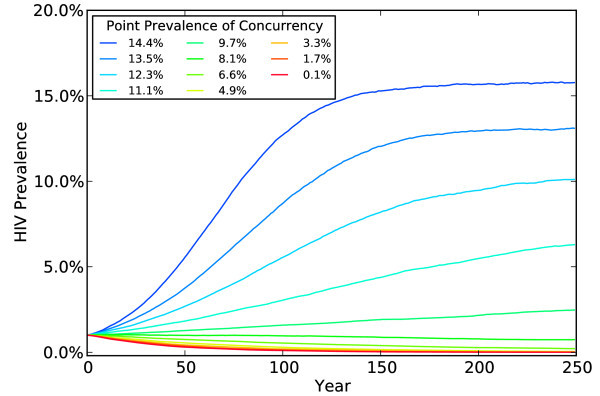
**HIV prevalence at different levels of concurrency from 0 to 250 years with no coital dilution**.

We present simulations to test the robustness of our results by successively modelling lower degrees of coital dilution. Table 2 reports the results of simulations with coital dilution set at 55%, 35%, 25% and 15%. We also include a set of simulations in the last rows of Table 2 with no coital dilution, that is, when daily infection risks in non-primary partnerships are identical to those in primary partnerships. With no coital dilution, our results reproduce those of Eaton *et al*. (Compare our Figure 2 to Figure 1(b) in [9].)

With 55% coital dilution, just as at 75%, HIV epidemics progress to extinction more rapidly at higher levels of concurrency than at lower levels. By reducing coital dilution to the 35% level, we find that concurrency slows rather than accelerates the process of epidemic extinction, but the epidemic still moves to extinction at all levels of concurrency. When we reduce coital dilution to the 25% level (so that daily infection risk in non-primary partnerships is only 25% less than in primary partnerships), we see a spreading epidemic at the highest level of concurrency (when 14% of the population has at least one concurrent partner), but epidemics moving to extinction at lower levels of concurrency.

When we further reduce coital dilution to the 15% level, we finally produce outcomes resembling those of Eaton *et al*. Even at that minimal level, however, coital dilution substantially moderates the impact of concurrency on HIV prevalence. For example, Eaton *et al *found that in 25 years, HIV prevalence rises from 1% to 2.42% at the highest level of concurrency (14% point prevalence). With only 15% less sex in non-primary partnerships, however, our model produces in 25 years only one-third the increase in HIV prevalence that Eaton *et al *found. Thus, even at implausibly high levels of concurrency, *any *considered degree of coital dilution produces substantial erosion in the ability of concurrency to spread HIV.

Another way to look at these results is to find how high concurrency must be to avoid epidemic extinction. The percentages in Table 2 in boldface type are ones without epidemic extinction and are located in the lower right corner of the table. Eaton *et al *found that with point prevalence of concurrency below 8%, HIV epidemics become extinct. Recall that no nationally representative survey in Africa in the past 20 years using currently accepted questionnaire design has found point prevalence of concurrency as high as 8%. We find that by assuming only a 15% reduction in daily infection risks for non-primary partnerships, that threshold rises from 8% to 11% point prevalence. When coital dilution reaches 25%, any point prevalence of concurrency lower than 14% produces epidemic extinction. At any higher degree of coital dilution, HIV epidemics progress to extinction at all considered levels of concurrency.

## Discussion

Our simulation includes both the network and the coital dilution effects of concurrency on HIV incidence, and it shows that heterosexual HIV epidemics quickly become extinct even at high simulated levels of concurrency. Researchers who model the effects of concurrency on HIV epidemic dynamics have ignored or inadequately accounted for coital dilution. In response to the available empirical evidence on coital frequency in non-primary partnerships, we distinguish between primary and non-primary partnerships by assigning lower stage-specific daily infection risk in non-primary partnerships. The available evidence, while admittedly thin, accords with our intuition that people do not typically increase their total sexual activity in proportion to the number of partners. Drawing on the work of Morris, Epstein and Wawer [10], our focal simulation assumes 75% coital dilution (so that daily infection risks in non-primary partnerships are 25% of those in primary partnerships).

At that level of coital dilution, epidemics progress rapidly to extinction at all considered levels of concurrency. The higher the level of concurrency, the more rapid is the progression to extinction. Eaton *et al *find that HIV epidemics progress to extinction when point prevalence of concurrency is 8% or lower. We find that with even modest degrees of coital dilution, that threshold quickly rises. Our sensitivity analysis demonstrates that even with only a 35% reduction in daily infection risks in non-primary partnerships due to coital dilution, HIV epidemics progress to extinction at all considered rates of concurrency.

Other researchers have also emphasized the importance of coital frequency in concurrent partnerships in explaining regional variations in HIV prevalence. Reniers and co-authors [17-19] argue that polygynous marriages protect against HIV at the population level, in part due to coital dilution. Our results show that in the presence of coital dilution, all concurrent partnerships, even if not sanctioned by law, religion, or custom, can be protective against HIV, not just polygynous marriages.

Although our results show a far smaller effect of concurrency on HIV epidemics than other modellers have found, we suspect that our simulations actually overstate the importance of concurrency. First, Additional File 2 explores additional reasons why Eaton *et al*'s model may substantially overstate concurrency's impact on HIV prevalence. Since our model is equivalent to theirs except for the addition of coital dilution, our results correspondingly overstate concurrency's role. Second, we assume in the model that daily infection risks are the same in all non-primary partnerships (rather than falling as successive partners are added) and that adding a second partner does not reduce coital frequency in a primary partnership. We suspect that neither of those assumptions is correct, in which case, our simulations overstate average coital frequency for all concurrent partnerships and our results systematically understate the coital dilution effect. Thus, our findings would overstate the impact of concurrency on HIV.

Limitations of the data also raise the possibility that our focal simulation might understate the impact of concurrency on HIV epidemics. Our estimate that coital frequency in non-primary partnerships is at least 75% lower than in primary partnerships is based on respondents' statements about the number of sex acts in those partnerships in the previous year [10]. If non-primary partnerships were more likely than primary partnerships to have begun and/or ended in that year, and if for some reason the data were not adjusted to account for the shorter duration, then some of the difference in reported median annual coition could have resulted from shorter average partnership duration rather than lower coital frequency during partnerships. Note that the mean overlap of partnerships in the different gender/location groups in Morris *et al *was just over two years in two groups, around five years in three groups, and over eight years in the remaining group. Given those extensive overlaps, the problem of different partnership duration is unlikely to have produced serious bias. Nevertheless, our sensitivity analysis shows that our results are robust even if our focal simulation substantially overestimates the degree of coital dilution.

## Conclusions

Coital dilution means that as levels of concurrency rise in a population and other sexual behaviours do not change, the number of sex acts in the population declines. That is so because individuals on average do not simply scale up their sexual activity as they take on additional partners. The falling number of sex acts reduces the number of times that HIV can be transmitted and consequently slows the spread of the virus. When even implausibly modest degrees of coital dilution are built into a model of sexual networks and HIV epidemic dynamics, modelled HIV epidemics move rapidly to extinction. Our work shows that in order to contribute usefully to the investigation of HIV prevalence and sexual network dynamics, simulation models must incorporate realistic degrees of coital dilution.

These findings have two implications. First, in contrast to the prior simulation literature, we have shown that there is no basis for featuring concurrency *per se *(as opposed to multiple partnerships in general) in any HIV-prevention message in sub-Saharan Africa or anywhere else. Second, our results indicate that concurrency cannot explain the extraordinarily high prevalence of HIV in sub-Saharan Africa, even if it could be shown that concurrency was more prevalent there. (See [26], which shows that concurrency is not especially prevalent in the region.) Evidence does not support the notion that differences in sexual behaviour are enough to explain Africa's hyper-epidemics of HIV [27-29]. Researchers should look for other drivers of the HIV epidemics in Africa.

## Competing interests

The authors declare that they have no competing interests.

## Authors' contributions

LS, AI and ES collaborated in posing the research topic, developing the modelling strategy, parameterizing the model, and drafting the manuscript. AI wrote the Python simulation code and managed the simulations. All authors have read and approved the final manuscript.

## Authors' information

Larry Sawers is Professor of Economics at American University. Alan G Isaac is Associate Professor of Economics at American University. Eileen Stillwaggon is Professor of Economics and Harold G. Evans-Eisenhower Professor at Gettysburg College.

## Supplementary Material

Additional file 1 **The assumption of constant coital frequency **[34-38]. Click here for file

Additional file 2 **Overstating the importance of concurrency in Eaton *et al ***[39]. Click here for file
