# Supplementary material for: HIV and concurrent sexual partnerships: modelling the role of coital dilution
Source: J Int AIDS Soc. 2011 Sep 13;14:44. doi: 10.1186/1758-2652-14-44 (PMC3182950; doi:10.1186/1758-2652-14-44)
Supplement: Additional file 1 — The assumption of constant coital frequency [34-38]. [file 1758-2652-14-44-S1.DOC]

**Additional File 1. The assumption of constant coital frequency**

By assuming the same daily transmission rate in all partnerships, Morris and Kretzschmar [2] implicitly assume the same but unspecified per-act transmission rate and the same but unspecified coital frequency. Otherwise, the transmission rate and coital frequency would have to vary inversely – in the formal mathematical sense – since their product (the daily transmission rate) is a constant. There is no evidence for such an inverse relationship, and Morris and Kretzschmar do not propose one. The only plausible understanding of Morris and Kretzschmar’s constant daily transmission rate is that it implicitly assumes fixed coital frequency. Essentially identical reasoning applies to Eaton *et al* [9], except in place of a single daily transmission rate, there are different transmission rates for each of four stages of infection. Again, unless they assume a fixed coital frequency at each stage of infection, they would have to assume that same inverse relationship. Hence, Eaton *et al* must implicitly assume fixed coital frequencies at each stage. It follows that Eaton *et al*’s use of the same daily risk of infection (at each stage of infection) for all partnerships is inconsistent with the evidence showing that coital frequency is lower in non-primary partnerships.

While there is no evidence for an inverse relationship between coital frequency and per-act transmission and we know of no one who suggests that one exists, Johnson *et al* [8] assert that per-act transmission rates are a negative function of the cumulative number of sex acts in a partnership. They use that assertion to justify much higher per-act transmission rates in non-spousal (though not necessarily concurrent) partnerships than in spousal partnerships in their model, which is almost the opposite of coital dilution. Not surprisingly, their model shows that most HIV transmission in SSA occurs in non-spousal partnerships.

They cite three articles [34-36] to support their assertion that there is “substantial evidence that the probability of HIV transmission per act of sex reduces as the cumulative number of sex acts with the infected partner increases” (page 302). One of the three articles they cite, however, provides no support for their assertion. Downs *et al* discuss many reasons why transmission efficiency might vary within a partnership (including sexually transmitted coinfections, condom use, and stage of infection), but never suggest that it is a function of cumulative sex acts [34].

The other two sources that Johnson *et al* cite were both published in 1990. The variation in transmission rates by stage of infection was not well understood at the time and the research presented in the articles did not attempt to avoid confounding from the higher infectivity during primary infection. Furthermore, the rate at which partners become infected can vary with partnership duration, not because the per-act transmission rate is a function of time, but because of differential infectiousness of the transmitting partners and susceptibility of the acquiring partners [37]. That would explain a high risk of seroconversions in the early days of some partnerships and a far longer time to seroconversion in other partnerships. Mathematical modelling has confirmed how variations in host susceptibility produce falling HIV incidence over time [38].

We are not aware of any researcher, including the authors of the three articles that Johnson *et al* cite, who in the past 15 years has found evidence for a causal connection between transmission rates and either cumulative coition or average coital frequency or thought the notion worthy of exploration. In sum, we find the evidence for Johnson *et al*’s assertion unconvincing and unsupported by current research. Thus, we accept the conventional presumption that the heterosexual transmissibility of HIV does not vary systematically with “the cumulative number of sex acts” of which Johnson speaks, nor with coital frequency.
