# Supplementary material for: HIV and concurrent sexual partnerships: modelling the role of coital dilution
Source: J Int AIDS Soc. 2011 Sep 13;14:44. doi: 10.1186/1758-2652-14-44 (PMC3182950; doi:10.1186/1758-2652-14-44)
Supplement: Additional file 2 — Overstating the importance of concurrency in Eaton et al [39]. [file 1758-2652-14-44-S2.DOC]

**Additional File 2. Overstating the importance of concurrency in Eaton *et al***

There are several reasons why Eaton *et al*’s model [9] overstates the impact of concurrency on HIV epidemics. First, the model assumes that men and women’s rates of concurrency and daily infection risk are the same despite the evidence to the contrary. The evidence indicates that coital dilution is far more pronounced among women than among men [10]. Gender asymmetry in rates of concurrency, per-act transmission rates, and degree of coital dilution weakens the ability of concurrency to spread of HIV. When we incorporate gender asymmetry in rates of concurrency into our model, we find that it reduces the impact of concurrency on the spread of HIV.

Second, Eaton *et al* [9] assume a daily transmission rate that is the same throughout the 88-day period of primary infection. Pilcher *et al* (Figure 2 in [39]) chart viral load during viremia, showing that it peaks sharply in the first weeks after infection. That suggests that Eaton *et al*’s daily transmission rate is too low in the earliest days of primary infection and too high later on. Eaton *et al* show that a staged transmission rate produces a slower growth in HIV and lower ultimate HIV prevalence than does a constant transmission rate. That result suggests that a finer-grained staging of infection risk that allowed the peaking of infectivity in the early days of the primary infection period would further reduce the impact of concurrency. With infrequent sex in non-primary partnerships and a narrower window of peak infectivity, the risk of transmission in those partnerships might be lower than implied by Eaton *et al*’s formulation.

Third, Hollingsworth *et al* say that their estimates of infection risk were “higher than expected” [24]. They say that “comparison [of our results] with transmission rates as a function of viral load estimated elsewhere indicates that the transmission rate during primary infection is significantly higher than would be expected on the basis of the plasma viral loads observed during these periods . . .” (page 689). They make the same assertion about infection risk during the late stages of the infection (page 690). They also suggest that “coinfection with other sexually transmitted pathogens” could have affected their results (page 690). In addition to sexually transmitted infections (STIs) that might have confounded Wawer *et al* and Hollingsworth *et al*’s data, there are numerous cofactor infections that are not STIs [29] and many non-sexual exposures other than injections or transfusions that could potentially increase observed transmission efficiency. If the daily transmission risks used by Eaton *et al* are too high, their simulations overstate the effect of concurrency on HIV prevalence.

Fourth, Eaton *et al* assume an average partnership duration of 200 days (following Morris and Kretzschmar [2]). There is evidence that average partnership duration in sub-Saharan Africa is much longer [3,10]. It is likely that at any given level of concurrency, the rate at which HIV spreads will vary inversely with partnership duration. Thus, the partnership duration parameter chosen by Eaton *et al* is likely to produce an overstatement of HIV prevalence at any level of concurrency.

Because of these problems with Eaton *et al*’s model, we take their work as an upper-bound estimate of the contribution of concurrency to HIV prevalence.
